# Effects of oral florfenicol and azithromycin on gut microbiota and adipogenesis in mice

**DOI:** 10.1371/journal.pone.0181690

**Published:** 2017-07-25

**Authors:** Rui Li, Hexing Wang, Qingfeng Shi, Na Wang, Zhijie Zhang, Chenglong Xiong, Jianxiang Liu, Yue Chen, Lufang Jiang, Qingwu Jiang

**Affiliations:** 1 Key Laboratory of Public Health Safety of Ministry of Education, School of Public Health, Fudan University, Shanghai, China; 2 School of Epidemiology and Public Health, Faculty of Medicine, University of Ottawa, Ottawa, Ontario, Canada; Universite Paris-Sud, FRANCE

## Abstract

Certain antibiotics detected in urine are associated with childhood obesity. In the current experimental study, we investigated two representative antibiotics detected in urine, florfenicol and azithromycin, for their early effects on adipogenesis, gut microbiota, short-chain fatty acids (SCFAs), and bile acids in mice. Thirty C57BL/6 mice aged four weeks were randomly divided into three groups (florfenicol, azithromycin and control). The two experimental groups were administered florfenicol or azithromycin at 5 mg/kg/day for four weeks. Body weight was measured weekly. The composition of the gut microbiota, body fat, SCFAs, and bile acids in colon contents were measured at the end of the experiment. The composition of the gut microbiota was determined by sequencing the bacterial 16S rRNA gene. The concentration of SCFAs and bile acids was determined using gas chromatography and liquid chromatography coupled to tandem mass spectrometry, respectively. The composition of the gut microbiota indicated that the two antibiotics altered the gut microbiota composition and decreased its richness and diversity. At the phylum level, the ratio of *Firmicutes*/*Bacteroidetes* increased significantly in the antibiotic groups. At the genus level, there were declines in *Christensenella*, *Gordonibacter* and *Anaerotruncus* in the florfenicol group, in *Lactobacillus* in the azithromycin group, and in *Alistipes*, *Desulfovibrio*, *Parasutterella* and *Rikenella* in both the antibiotic groups. The decrease in *Rikenella* in the azithromycin group was particularly noticeable. The concentration of SCFAs and secondary bile acids decreased in the colon, but the concentration of primary bile acids increased. These findings indicated that florfenicol and azithromycin increased adipogenesis and altered gut microbiota composition, SCFA production, and bile acid metabolism, suggesting that exposure to antibiotics might be one risk factor for childhood obesity. More studies are needed to investigate the specific mechanisms.

## Introduction

According to data released by the World Health Organization (WHO) in 2008, more than one billion adults worldwide were overweight, and among those individuals, 300 million adults were obese[1,2]. Obesity is not only prevalent in adults but also in children[3,4]. Obesity is related to specific major chronic diseases, such as type II diabetes mellitus, coronary heart disease, atherosclerosis, and nonalcoholic fatty liver disease[5]. However, the etiology of obesity is not fully understood. Recently, the gut microbiota was found to be closely correlated with metabolic and immune function and an important contributor to adipogenesis. For example, Ley et al. found that obesity was related to intestinal microorganisms, with a decreased ratio of *Bacteroidetes* to *Firmicutes* observed in obese mice compared to lean mice from the same brood[6]. Severe obesity and insulin resistance occurred in mice one week after *in vivo* inoculation of *Enterobacter cloacae B29* that could produce endotoxins[7]. The gut microbiota in human intestinal tracts was highly diverse in normal weight subjects compared with obese subjects, with *Firmicutes* more predominant in obese subjects [8]. Obese people with low microbial gene species richness tend to gain more weight with a high fat diet than obese people with high species richness[9].

The gut microbiota has a close relationship with the body's energy homeostasis and fat metabolism, but it also tends to be affected by factors such as breastfeeding, caesarean section, and dietary habits, as well as other factors [10,11]. Among them, exposure to antibiotics is a potential threat that cannot be ignored. Due to extensive use in humans and animals, antibiotics have frequently been found in aquatic environments and in food. Thus, they are listed as a class of emerging environmental pollutants [12]. Compared to other environmental pollutants, antibiotics can not only do direct harm to the human body but can also interfere with the human microbiome, possibly resulting in metabolic diseases, such as obesity[13]. For example, lab studies have found that certain antibiotics, such as vancomycin, penicillin and chlortetracycline, can alter the bacterial composition and endocrine activity of the gut microbiota, affect production of short-chain fatty acids (SCFAs) and the metabolism of bile acids, and increase body fat [14–18]. Several epidemiological studies have reported that antibiotic use in children was positively associated with obesity in children[19–22]. Recently, several studies have reported extensive exposure of school-aged children to antibiotics by measuring antibiotics in urine, and certain antibiotics, such as florfenicol and trimethoprim, were related to obesity [23,24].

Antibiotics have different effects on gut microbiota due to differences in their antimicrobial spectrum and thus differ in their capacity to interfere with fat metabolism. Florfenicol and azithromycin are two typical antibiotics frequently detected in urine, and they have a potential impact on fat metabolism, but there is still a lack of laboratory data regarding their effect on adipogenesis. Since florfenicol first came to the market in Japan in the 1990s, it has been used in a number of countries to treat bacterial disease in animals including cattle, pigs, chicken and fish. Florfenicol is an amphenicol antibiotic and has a broad spectrum of antimicrobial activity that includes a wide range of gram-positive and gram-negative bacteria and mycoplasma [25]. Because it is widely used in aquaculture, florfenicol is typically more abundant in water environments than other antibiotics. Azithromycin has relatively broad but shallow antibacterial activities. It inhibits some gram-positive bacteria, some gram-negative bacteria, and many atypical bacteria. Azithromycin is a second generation macrolide that is primarily used to treat respiratory tract and genital tract infections, and it has been recommended by a number of countries and regions as a first-line treatment for these types of infections [26]. To support the findings in human populations, the current study aimed to explore the effects of azithromycin and florfenicol on the gut microbiota and adipogenesis in mice.

## Materials and methods

### Mouse model, sample collection, and body measurement

The study was approved by the research ethics committee of Fudan University, and carried out in accordance with the relevant guidelines and regulations. To explore possible effects of early exposure to azithromycin and florfenicol on adipogenesis in mice, we chose to start the experiment when mice were four weeks old, which is in line with previous studies, because this time is considered to be a critical period for gut microbiota development in mice [14]. A total of 30 three-week-old C57BL/6 mice were obtained from the Animal Experimental Center of Fudan University. The mice were randomly divided into three groups (azithromycin, florfenicol, and control), and each group included ten mice (five males and five females). The mice were separately bred in polycarbonate cages based on sex and group. Before the experiment, the mice were acclimatized to standardized laboratory conditions at a temperature of 21±1°C, a relative humidity of 50±10%, and a 12 h light-dark cycle for one week. These conditions were maintained throughout the entire study. Antibiotics were orally administered in drinking water for four weeks starting the fourth week after birth. The drinking water was spiked with azithromycin and florfenicol at a concentration of 35 mg/L and 33 mg/L, respectively. We changed the solutions containing the antibiotics every day. According to the daily consumption by mice, the exposure dose was estimated to be 5 mg/kg/day for each of the two antibiotics[14]. Florfenicol and azithromycin dihydrate with a purity above 98% were purchased from Sigma-Aldrich Corporation (St Louis, MO, USA). During the experiment, no adverse events were observed. At the end of each week during antibiotic administration, the mice were weighed using an electronic scale. At the end of the fourth week during antibiotic administration, the body fat content of all mice was determined after excretion of feces and urine using a MesoQMR nuclear magnetic resonance analyzer for body composition analysis of conscious animals (Niumag Corporation). Mice were killed by CO_2_ inhalation and cervical dislocation. Then, mice were dissected, an incision was made in the colon with a sterile scalpel, and the contents of the colon were collected into sterilized centrifuge tubes using sterile forceps. All samples were immediately flash-frozen in liquid nitrogen and stored at -80°C until use.

### 16S rRNA gene sequencing and data processing

Total genomic DNA was extracted from thawed colon content samples using a Powersoil DNA Extraction Kit (MoBio, Carlsbad, CA, USA) in 96-well format, and the 16S rRNA gene was amplified with barcoded fusion primers targeting the V3, V4, and V5 regions. Amplicon pools were sequenced on a 2×150 bp Illumina MiSeq platform. Two reads were paired, and paired-end reads were assembled with an overlapping region of at least 20 bp guaranteed; reads containing N were removed. Primers and adaptor sequences, bases with a quality of less than 20 at both ends, and sequences with a length of less than 400 bp were excluded. After pairing the abovementioned sequences, high-quality sequences were classified into multiple operational taxonomic units (OTUs) according to sequence similarity (> 97%). To make the data more interpretable, we edited the OTUs according to their representation among the samples. We ranked the abundance of OTUs from high to low, and OTUs after the top 30 were classified into “Others”. This classification reduced the noise of amplicon datasets and avoided spurious associations when there was a preponderance of zero counts. Taxonomic assignment and diversity calculations were also conducted. The distance between samples was calculated using the evolution and abundance information in the sequence of each sample to reflect whether there was a significant difference in the microbial community between samples. Linear discriminant analysis effect size (LEfSe) was employed to detect significant differences in relative abundance of microbial taxa between groups at the species level.

### Analysis of SCFAs and bile acids in colonic contents

The SCFA standards included acetic acid (AA), propionic acid (PPA), isobutyric acid (IBA), n-butyric acid (NBA), isovaleric acid (IVA), n-valeric acid (NVA), and 2-ethylbutyric acid (EBA). The bile acid standards included cholic acid (CA), deoxycholic acid (DCA), chenodeoxycholic acid (CDCA), lithocholic acid (LCA), ursodeoxycholic acid (UDCA), hyodeoxycholic acid (HDCA), and two isotope-labeled bile acids (LCA-d4 and CA-d5). All these standards were obtained from Sigma-Aldrich Corporation, Dr. Ehrenstorfer (Augsburg, Germany), and Anpel Laboratory Technologies Inc. (Shanghai, China). The SCFAs and bile acids in the colon contents were determined in our lab using modified methods published previously [27].

For SCFAs, after 50.0 mg of the colonic contents was weighed and EBA was added as an internal standard, the sample was completely homogenized with 1.0 ml of 0.5 M oxalic acid for 5 min and centrifuged at 8000 r/min for 10 min. The supernatant was filtered with a 0.22 μm nylon membrane, and 1 μl of the filtrate was analyzed using a capillary gas chromatography instrument equipped with a flame ionization detector (FID) (GC-2010 Plus, Shimadzu). The SCFAs were separated on a fused-silica capillary column with a free fatty acid phase (DB-FFAP) and dimensions of 30 m × 0.53 mm i.d. coated with 1.0 μm film thickness. Nitrogen was used as the carrier gas at an initial flow rate of 6.44 ml/min in the constant pressure mode. The initial oven temperature was 50°C. This temperature was maintained for 1.0 min, raised to 180°C at 20°C/min, held for 1.0 min, and then increased to 200°C at 20°C/min and held for 2 min. Glass wool was inserted into the glass liner of the injection port, and the split injection mode was used with a split ratio of 30 to 1. The FID temperature and injection port was 240 and 200°C, respectively. The flow rates of hydrogen, air and nitrogen that composed the gas for the FID were 40, 400 and 30 ml/min, respectively. The SCFAs were quantified using the internal standard curve method.

For bile acids, after 50.0 mg of colonic content was weighed and isotope-labeled internal standards (LCA-d4 and CA-d5) were added, the bile acids were completely homogenized with 1 ml of 0.2 M NaOH for 20 min and centrifuged at 8000 r/min for 10 min. The extraction step was repeated three times, and the supernatants were combined. After the Oasis Prime HLB cartridge (60 mg/3 cc, Waters) was conditioned with 2 ml of methanol and 2 ml of water, the combined supernatants were loaded. The cartridge was washed with 2 ml of 20% methanol in water solution, and bile acids were eluted with 3 ml of acetonitrile:methanol (90:10) containing 1% formic acid. The eluent was evaporated with a weak nitrogen flow, and the bile acids were reconstituted with 0.5 ml of 50% methanol water solution. After the reconstituted solution was filtered through a 0.22 μm nylon membrane, 10 μl of the filtrate was analyzed using ultra-performance liquid chromatography coupled with high-resolution quadrupole time-of-flight mass spectrometry (SYNAPT G2, Waters Micromass, Manchester, UK). Bile acids were separated on an analytical column (Acquity UPLC HSS T3 column, 100 mm × 3.0 mm × 1.8 μm) at a column temperature of 50°C with a mobile phase of methanol and water containing 0.1% formic acid at a flow rate of 0.55 ml/min. The linear elution program was as follows: from 0 to 3.00 min, increased to 35% from 5% methanol; from 3.00 to 6.00 min, increased to 65% methanol; from 6.00 to 8.00 min, increased to 80% methanol; from 8.00 to 9.00 min, increased to 90% methanol; from 9.00 to 10.00 min, increased to 95% methanol; from 10.00 to 10.50 min, increased to 98% methanol; from 10.50 to 11.00 min, decreased to 5% methanol and maintained until 12.00 min. For mass spectrometry, nitrogen and argon were used as the desolvation and collision gas, respectively. The flow rate of the desolvation gas was set to 800 L/h with a temperature of 400°C. The capillary voltage was set to 2.8 kV, the cone gas was set to 30 L/h, the source temperature was set to 110°C, and the sampling cone voltage was set to 30 v. The bile acids were quantified using the isotope-dilution method, and the calibration curve was based on the peak area of the extracted precursor ion chromatogram for each bile acid from the total ion chromatogram at a mass window of 0.02 Da.

### Statistical analysis

Quantitative data are reported as the mean ± standard error. The Kolmogorov—Smirnov D test was used to ascertain the normality of the data. Statistical differences in body composition and the colonic SCFA and bile acid content between the groups were tested with one-way analysis of variance (one-way ANOVA) and multiple t-tests with Bonferroni correction for continuous variables. All analysis was conducted for males and females separately. A p-value less than 0.05 was considered statistically significant. All statistical analyses were performed using R 3.1.1 software[28]. Reads were assembled using PANDAseq (v. 2.7) [29]. Trimmomatic (v. 0.30) was used to filter primers and adapter sequences[30]. USEARCH (v. 8.0) was employed to pair assembled and filtered reads[31]. The QIIME pipeline with RDP classifier Bayesian algorithm was used for taxonomic assignment with the SILVA_119 16S rRNA database [32,33]. OTU classification, UniFrac analysis, and calculation of diversity metrics were also conducted with QIIME pipeline. Linear discriminant analysis effect size (LEfSe) was conducted using Galaxy Online software [34,35].

## Results

### Body weight and composition

**Fig 1:**
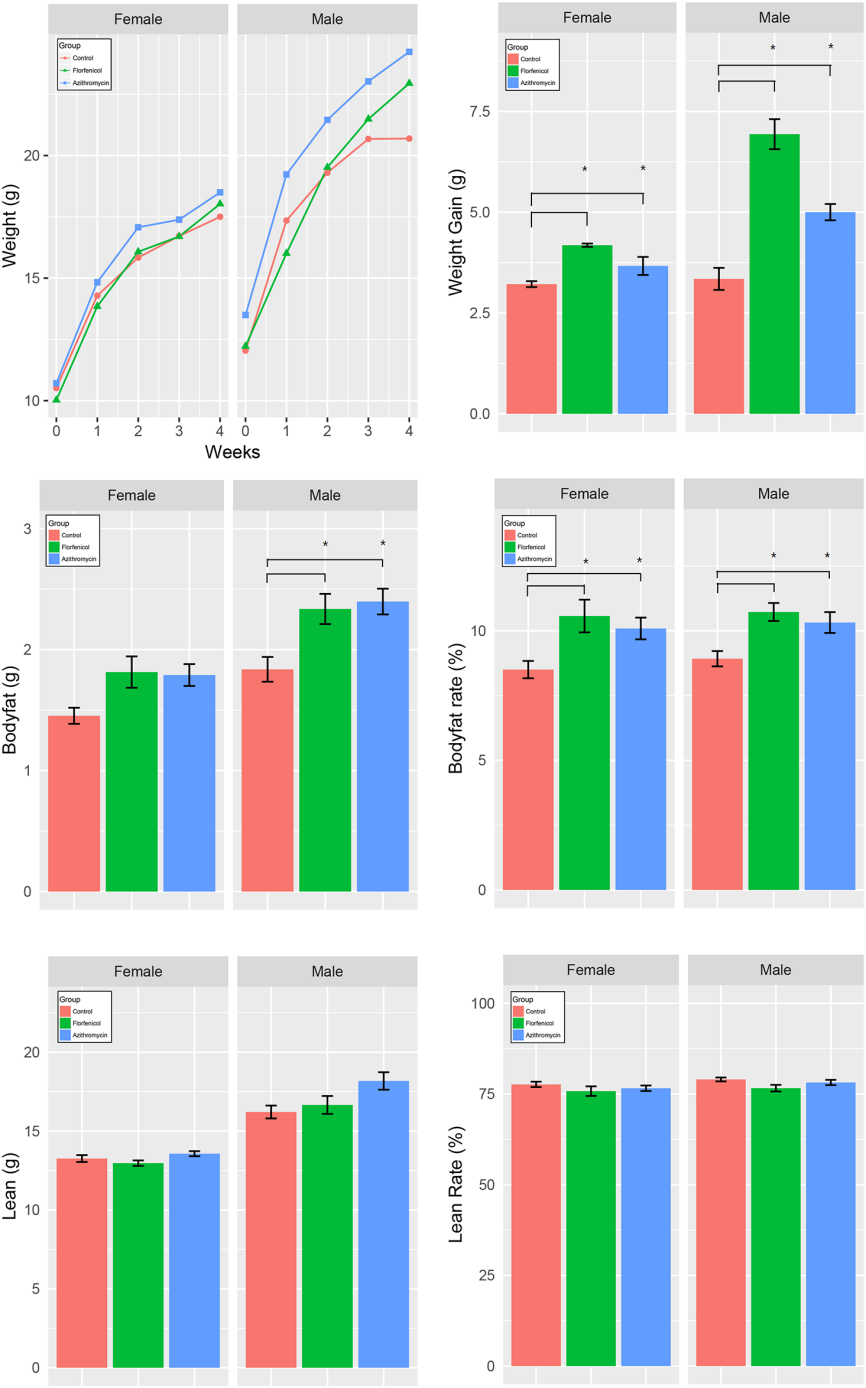
The weight change within four weeks and body composition after four weeks. One-way analysis of variance (one-way ANOVA) and multiple t-tests with Bonferroni correction were employed to measure differences in weight gain, body fat (g), body fat rate (%), lean weight (g), and lean rate (%) between the three groups. * indicates that the difference between the two line-linked groups was statistically significant (p < 0.05).

As shown in Fig 1, there was no significant difference in weight among the 3 groups at the beginning of the experiment. The antibiotic groups gained more weight than the control group during the 4-week period, especially the males. The weight gains in the florfenicol group between week 4 and week 1 were larger than in the azithromycin group. The average percent body fat in the florfenicol group (10.62% ± 0.34%) and azithromycin group (10.20% ± 0.28%) was significantly higher than that in the control group (8.73% ± 0.22%). The differences in body fat between the antibiotic groups and the control group were larger in males than in females. The difference in weight gain between the florfenicol group and the control group was also larger in males than in females. The absolute value or rate of lean mass was similar for antibiotic-treated and control mice.

### General OTU information

A total of 14,226,262 16S rDNA sequence reads from V3, V4, and V5 regions were obtained from the 30 samples. The number of sequence reads for each sample ranged from 368,026 to 620,382 with an average of 474,208. The average length of the sequence reads was 445 bp. The taxon abundance of each sample was generated into 12 phyla, 23 classes, 33 orders, 61 families, 125 genera and 255 species. Overall, twelve phyla were identified in the study groups. Among them, five phyla were commonly found in each group, and three phyla were dominant at > 1% concentration but varied in relative abundance with sex and antibiotic treatment. The majority of sequencing reads at the phyla level were *Firmicutes*, *Bacteroidetes*, and *Proteobacteria*, which dominated the bacterial community in each of the studied groups. A total of 61 families were detected. Among them, nine families were commonly present, and *Lachnospiraceae*, *Ruminococcaceae*, and *Helicobacteraceae* were the dominant families detected. A total of 125 OTUs were identified at the genus level. The highest number of genera (125) was detected in the male control group, whereas the lowest (76) was found in the female mice treated with azithromycin. Antibiotics significantly decreased the number of genera in each of the antibiotic treatment groups. A total of 255 different species were found, and the highest number of species presented in the control group.

### Diversity of gut microbiota

**Fig 2:**
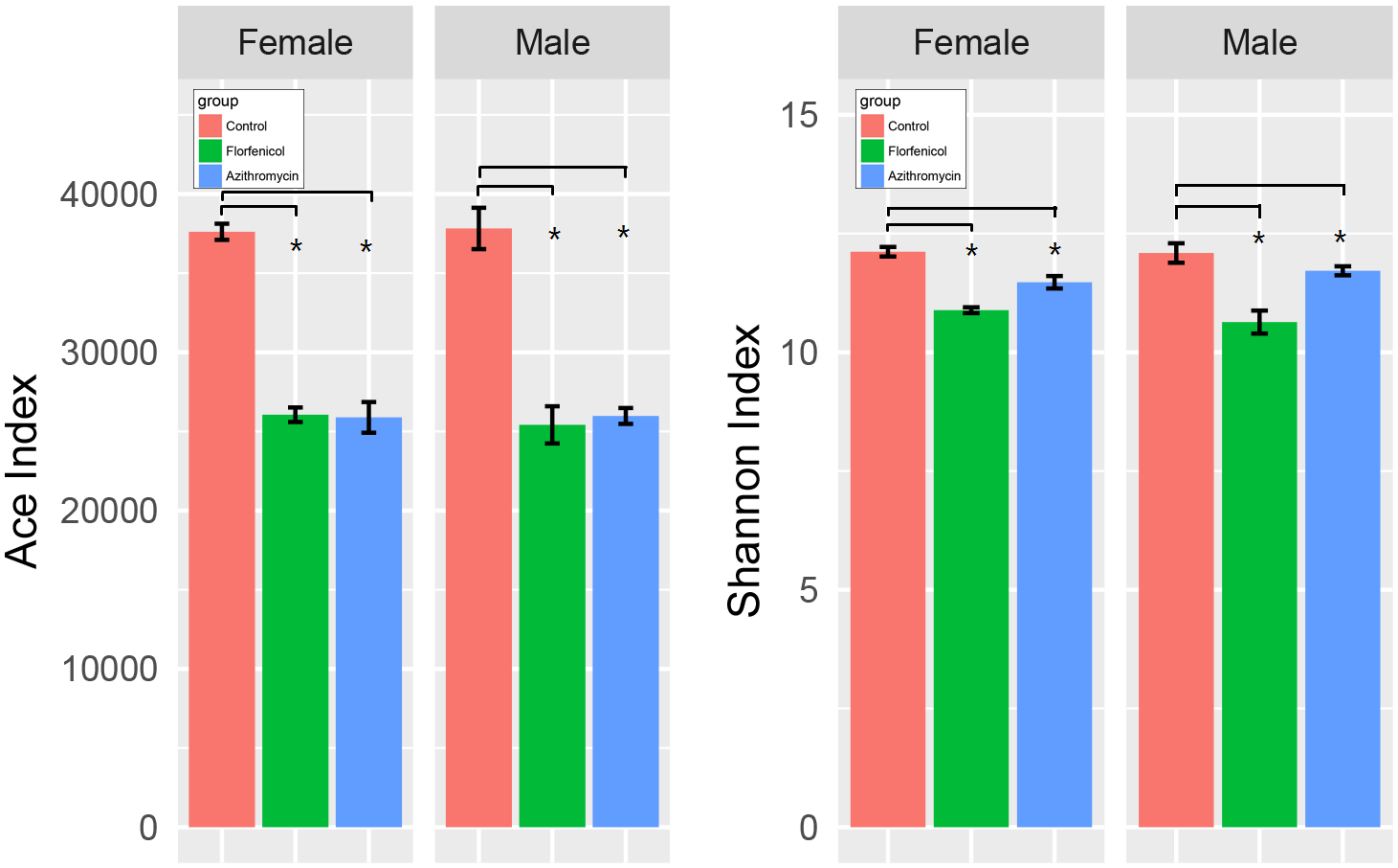
Ace index and Shannon index in the samples from the normal and antibiotic-treated groups. One-way analysis of variance (one-way ANOVA) and multiple t-tests with Bonferroni correction were employed to measure differences between the three groups. The ACE index represents the community richness of the gut microbiota, and the Shannon index represents the community diversity of the gut microbiota. * indicates that the difference between the two line-linked groups was statistically significant (p < 0.05).

**Fig 3:**
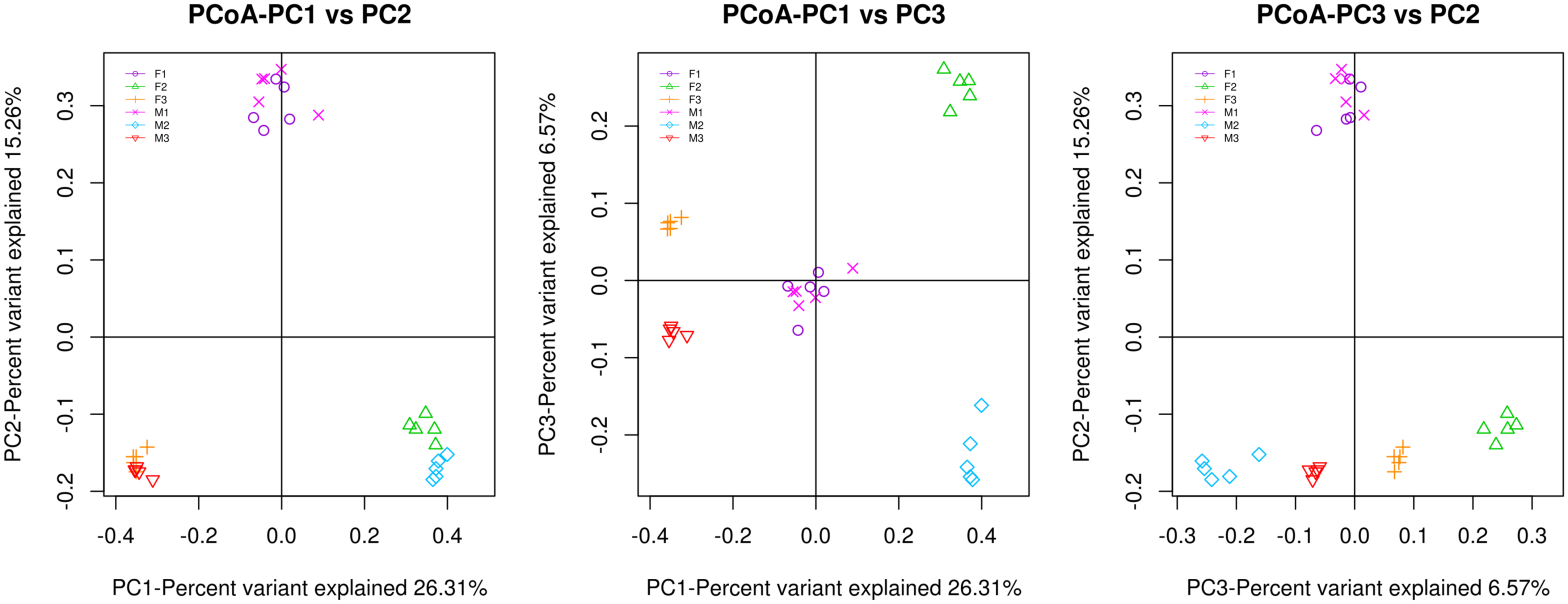
The multivariate principal coordinate data analysis of the groups. F1, female control group; F2, female mice treated with florfenicol; F3, female mice treated with azithromycin; M1, male control group; M2, male mice treated with florfenicol; M3, male mice treated with azithromycin. Each group has ten mice (5 male and 5 female subjects). PC1 and PC2 are the two principal coordinate components. PC1 indicates the maximum possible interpretation of the main components of the data variations. PC2 represents the largest proportion of interpretation of remaining variations, etc.

There are two types of alpha diversity indexes, community richness indexes (Chao, ACE) and community diversity indexes (Shannon). Fig 2 shows that the control group had higher richness and diversity index levels than the antibiotic groups (both males and females), and there were no significant sex-related differences. Good’s coverage, a measure of sampling completeness, ranged between 91.86% and 96.89% at a 97% similarity level. Beta diversity reflects diversity differences among samples. UniFrac principal coordinates analysis (PCoA) of 15,034 OTUs (grouped at 97% sequence similarity) showed a clear separation between the control and antibiotic samples using an unweighted analysis (Fig 3). The percentages of variation represented by PC1 and PC2 were 26.31% and 15.26%, respectively. In the two antibiotic groups, the male and female groups were also well separated.

### Composition of gut microbiota

**Fig 4:**
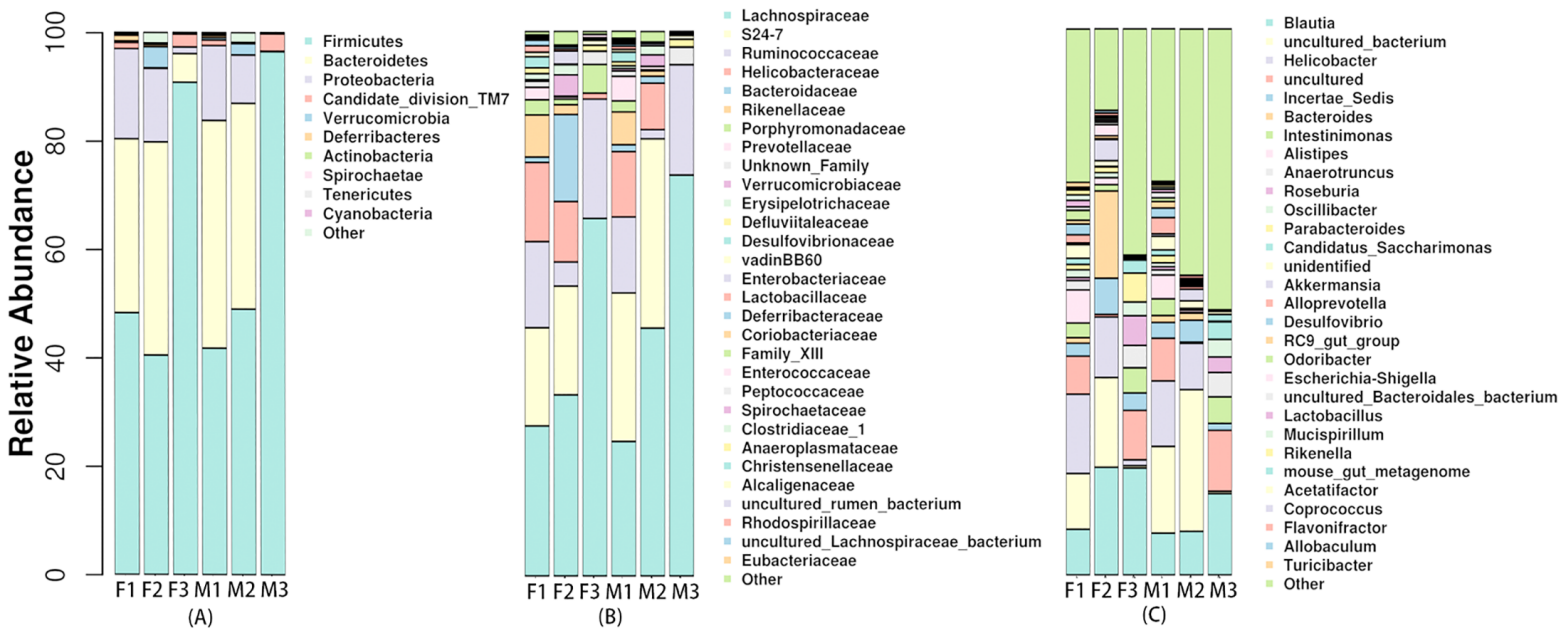
Microbial distribution at three different levels in samples from the normal and antibiotic-treated groups. (A) Taxonomic distribution of the top 10 phyla; (B) Taxonomic distribution of the top 30 families; (C) Taxonomic distribution of the top 30 genera. Abbreviations are the same as those described for Fig 3. Each group has ten mice (5 male and 5 female subjects).

There were substantial variations in the composition of gut microbiota associated with antibiotics and sex. Scoring and ranking were carried out for these differences. In females, Fig 4a shows that at the level of phylum, the relative abundance of *Firmicutes* increased and the relative abundance of *Bacteroidetes* and *Proteobacteria* decreased significantly in the azithromycin group compared with the control group. The relative abundance of *Verrucomicrobia* increased and that of *Deferribacteres* decreased significantly in the florfenicol group compared with the control group. In males, the relative abundance of *Firmicutes* increased while that of *Bacteroidetes* decreased to nearly zero in the azithromycin group compared with the control group. For florfenicol-treated mice, the relative abundance of *Firmicutes* was slightly higher in males than in females. For azithromycin-treated mice, females had higher relative abundance levels of *Bacteroidetes* and *Proteobacteria* and a lower relative abundance level of *Firmicutes* than males.

At the family level, Fig 4b shows that in females the relative abundance of *Lachnospiraceae*, *Ruminococcaceae*, and *Porphyromonadaceae* increased, while that of *Helicobacteraceae*, *Rikenellaceae*, and *Bacteroidaceae* decreased significantly in the azithromycin group compared with the control group. In the florfenicol group, the relative abundance of *Bacteroidaceae* and *Verrucomicrobiaceae* increased, whereas the relative abundance of *Ruminococcaceae*, *Helicobacteraceae*, *Rikenellaceae* and *Porphyromonadaceae* decreased compared with the control group. After florfenicol treatment, males had a lower relative abundance of *Bacteroidaceae* than females. After azithromycin treatment, the relative abundance of *Porphyromonadaceae* decreased in males but increased in females compared with the control group.

At the genus level, Fig 4c shows that in females the relative abundance of *Blautia* and *Parabacteroides* increased, but that of *Helicobacter* and *Alistipes* decreased significantly in the azithromycin group compared with the control group. The relative abundances of *Blautia* and *Bacteroides* increased, while that of *Alistipes* decreased in the florfenicol group. In males, the relative abundance of *Parabacteroides* did not change significantly compared with the control group. Moreover, the relative abundance of *Blautia* and of *Bacteroides* was not notably different between the florfenicol and control groups.

**Fig 5:**
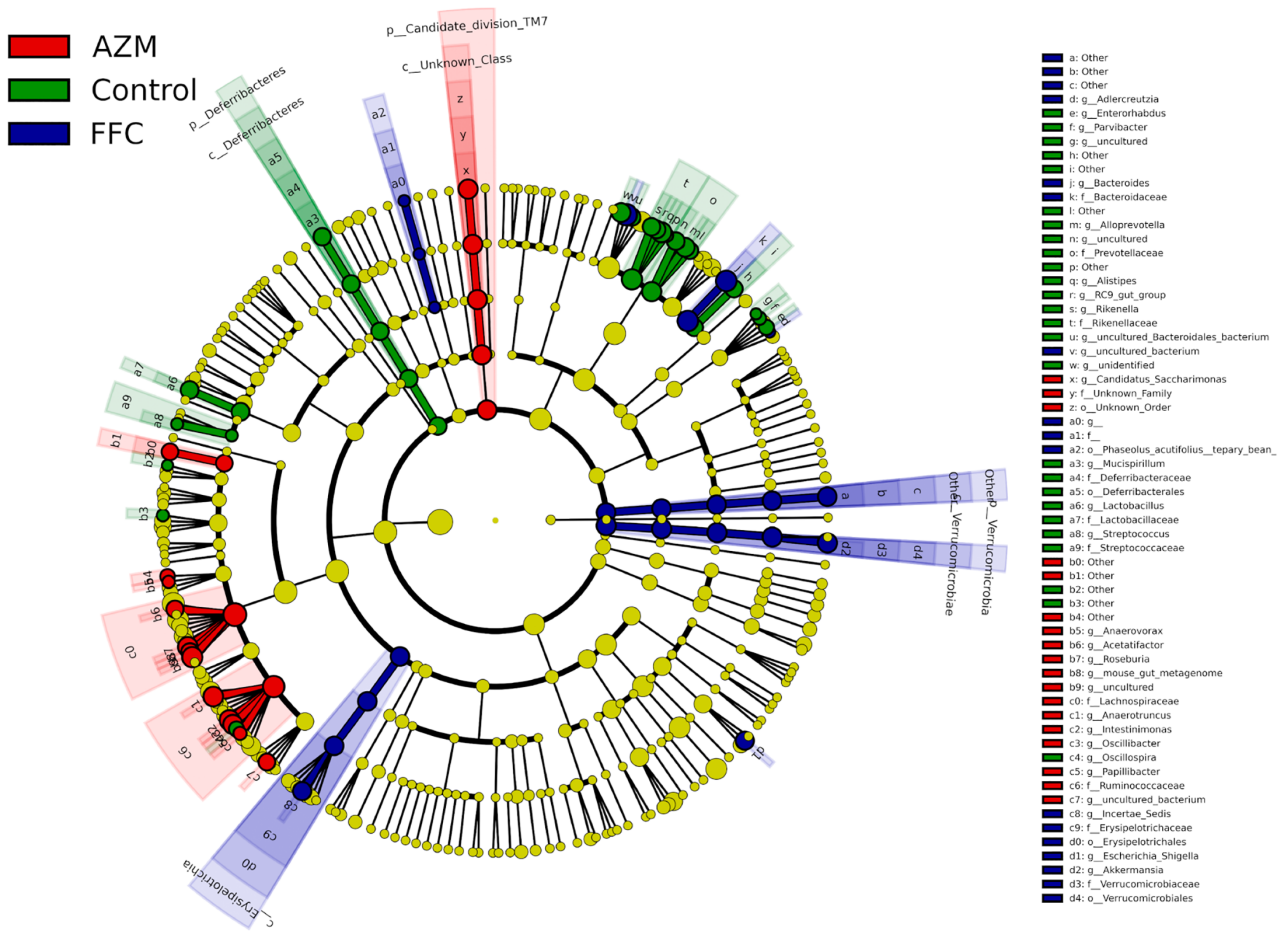
LEfSe results in the three groups, representing the relevant features on taxonomic trees. AZM, mice treated with azithromycin; FFC, mice treated with florfenicol. Due to the length of bacterial names, abbreviations are employed under the family level. Each group has 10 mice (5 male and 5 female subjects).

The LEfSe determines the features most likely to explain differences between groups by coupling standard tests for statistical significance with additional tests encoding biological consistency and effect relevance. At the genus level, *Christensenella*, *Gordonibacter*, and *Anaerotruncus* decreased in the florfenicol group; *Lactobacillus* decreased in the azithromycin group; *Alistipes*, *Desulfovibrio*, *Parasutterella* and *Rikenella* declined in both the antibiotic groups; and the decrease of *Rikenella* in the azithromycin group was particularly noticeable. Furthermore, LEfSe analysis identified other representative species of each group as biomarkers to distinguish different groups (Fig 5).

### SCFAs and bile acids

**Fig 6:**
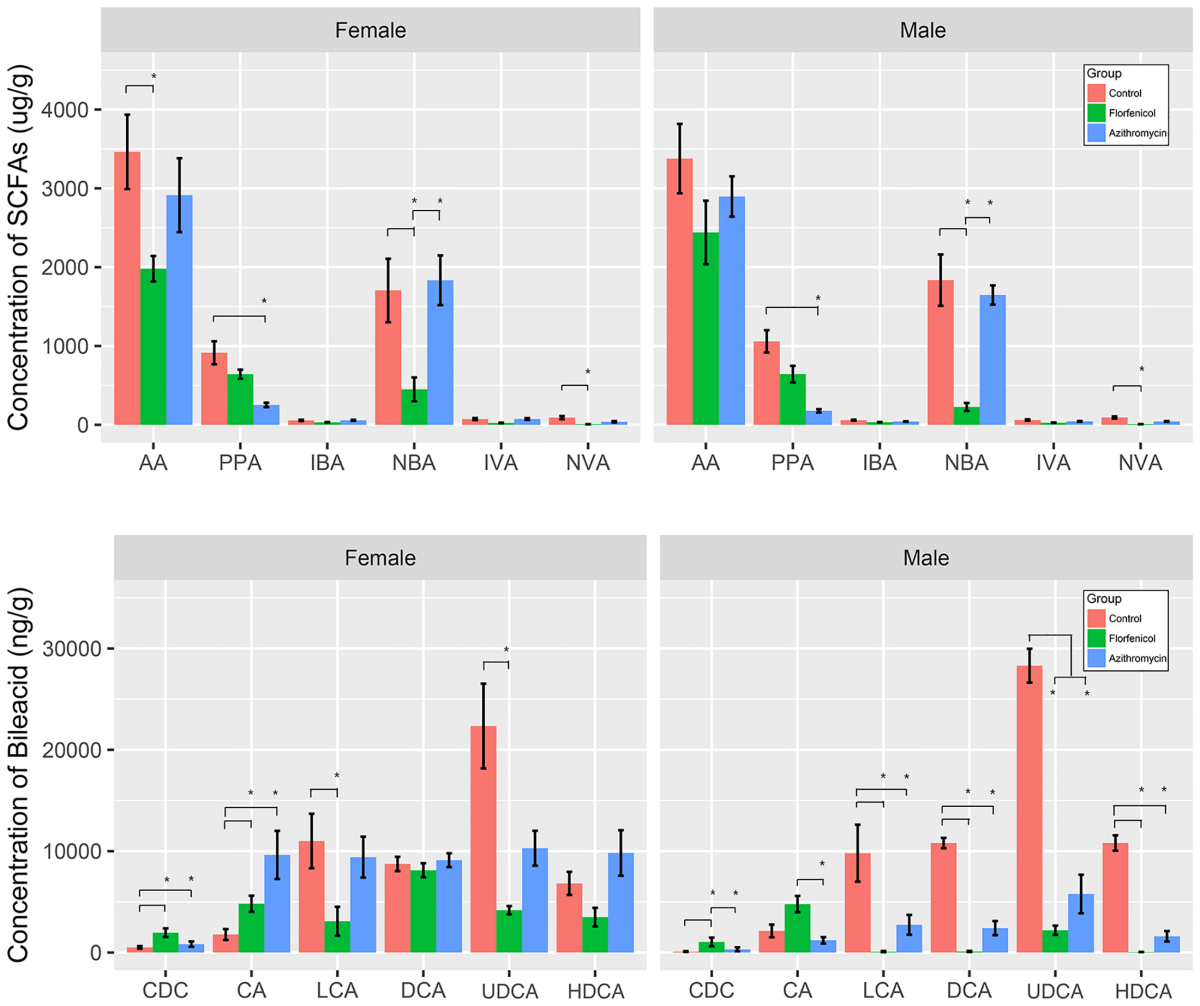
Concentration of SCFAs and bile acids in samples from the normal and antibiotic-treated groups. One-way analysis of variance (one-way ANOVA) and multiple t-tests with Bonferroni correction were employed to measure the concentration differences in bile acids and SCFAs between the three groups. * indicates that the difference between the two line-linked groups was statistically significant (p < 0.05).

As shown in Fig 6, the two antibiotics decreased the concentration of some SCFAs in the colonic contents, and florfenicol showed a stronger effect compared with azithromycin. Florfenicol decreased the concentration of some SCFAs in the colonic contents, especially AA, NBA, and NVA, with a 10-fold lower concentration than that in the control group. However, for azithromycin, only PPA and NVA showed decreased concentrations. There were no significant differences between the male and female groups.

In males, the mice treated with florfenicol had a higher concentration of CDCA and lower concentrations of LCA, DCA, UDCA and HDCA compared with the control group. The concentrations of all bile acids except CDCA and CA in the mice treated with azithromycin decreased compared with the control group, and the mice treated with azithromycin showed no significant difference compared with those treated with florfenicol. There were some differences between the male and female groups. Compared with females, males had lower concentrations of all bile acids in the azithromycin group and lower concentrations of secondary bile acids (LCA, DCA, UDCA, HDCA) in the florfenicol group.

## Discussion

In this study, azithromycin and florfenicol were found to increase adipogenesis in mice and alter the overall abundance, diversity and composition of the gut microbiota, the production of SCFAs, and the metabolism of bile acids in colonic contents. The changes in gut microbiota showed some antibiotic- or sex-specific differences. To the best of our knowledge, this is the first time the effects of azithromycin and florfenicol on gut microbiota composition and adipogenesis in mice have been explored.

We observed that antibiotics disturbed the gut microbiota composition. Most previous studies support these finding. For example, oral administration of amoxicillin, cefotaxime, and vancomycin decreased the abundance and altered the gut microbiota composition in rats[17]. The gut microbiota diversity indicated by the Shannon index was lower in both the antibiotic groups than in the control group, suggesting that the antibiotics reduced not only the richness but also the diversity of the gut microbiota, which is in line with results from previous studies[17,36]. Our study also found that azithromycin decreased the relative abundance of *Bacteroidetes* and *Proteobacteria* and increased the relative abundance of *Firmicutes*, which corresponds with the results of most other studies [6,37]. However, in Khan’s study, the *Bacteroidetes/Firmicutes* ratio increased[36]. Differences in dose, type of mice and the duration of experiments may be the reason for this inconsistency. Azithromycin increased the relative abundance of *Parabacteroides* and decreased the relative abundance of *Desulfovibrio* in the female group, but the opposite results were observed in the male group. This might be related to the differences in endocrine activity and the genetic background between male and female mice and needs to be further explored.

Both florfenicol and azithromycin decreased the concentrations of SCFAs in the colons of mice, but some differences were seen between the two antibiotics. This may be related to the changes in relative abundance and number of SCFA-producing bacteria in the gut microbiota induced by these antibiotics. For example, the decrease in the relative abundance of dominant propionate-producing *Bacteroidetes* in the group treated with azithromycin could have caused a reduced concentration of propionate in the colonic sample[38]. However, the relative abundance of acetate-producing *Blautia* increased in all the antibiotic groups except for the group of male mice group treated with florfenicol, but the concentration of acetate in these groups did not increase[39]. This may be explained by the possibility that the decrease in other acetate-producing bacteria, such as *Ruminococcaceae*, or the effects of the decreased absolute amount of *Blautia* on the production of acetate exceeded the effect of the increased relative abundance of acetate-producing *Blautia* [38]. Moreover, the relative abundance of butyrate-producing *Roseburia* and *Anaerotruncus* increased in mice treated with azithromycin but not in mice treated with florfenicol, which might explain the discrepancy in butyrate concentration between the two experimental groups[40].

Some human studies reported a decreased concentration of SCFAs induced by antibiotics, which supports our findings. Høverstad et al. reported that oral intake of particular antibiotics reduced the concentration of SCFAs in fecal samples under a therapeutic dose[41]. Young also found that amoxicillin under a therapeutic dose decreased the concentration of butyrate in fecal samples from men[42]. However, Cho’s result was inconsistent with our findings; in his study, penicillin and vancomycin at an exposure dose of 1 μg per g body weight increased most of the SCFAs in mice[14]. The possible reason for this could be the different exposure dose and type of antibiotic. In our study, a therapeutic dose was used, which reduced the total number of SCFA-producing bacteria and then decreased the concentration of SCFAs in the gut.

We found that florfenicol and azithromycin increased the concentrations of primary bile acids in the colon but decreased the concentrations of secondary bile acids, suggesting down-regulation of the 7-dehydroxylation process. Some studies have also reported similar effects for other antibiotics, such as vancomycin. In a single-blinded randomized controlled trial with 20 male subjects, 7 days of vancomycin t.i.d. was found to decrease fecal secondary bile acids and increase primary bile acids in plasma and fecal samples under the therapeutic dose [43,44]. The 7-dehydroxylation activity of *Clostridium* and *Eubacterium* in the gut microbiota is the key process that turns primary bile acid into secondary bile acid [45]. However, these two bacterial genera had extremely low relative abundances in the gut microbiota of our experimental mice, suggesting there might be other undiscovered bacteria responsible for 7-dehydroxylation. For example, other Clostridium family microbiota, such as *Ruminococcaceae* and *Lachnospiraceae*, might share similar functions with *Clostridium scindens*, and the relative abundances of these bacteria were also decreased by florfenicol and azithromycin [46]. Another possible explanation could be that the florfenicol and azithromycin decreased the total level of gut microbiota in our study as indicated by the Chao and ACE index, and they reduced the number of all bacteria involved in secondary bile acid synthesis. Sayin et al. found that germ-free mice had a higher gene expression level of primary bile acid biosynthesis compared with normal C57BL/6 mice, which supports the above explanation[27]. Furthermore, there are other reactions related to bile acid metabolism, such as deconjugation, oxidation and epimerization, and *Bacteroidaceae* and *Lachnospiraceae* are the main taxa that participate in these reactions [47]. In a human study, after subjects were treated with rifaximin b.i.d. for 8 weeks, the relative abundance of *Bacteroidaceae* and *Lachnospiraceae* in fecal samples was negatively correlated with primary bile acids and secondary/primary bile acid ratios in serum samples[46]. This finding is consistent with our results, indicating that in addition to the 7-dehydroxylation process other reactions, possibly involving *Bacteroidaceae* and *Lachnospiraceae*, also affect the production of secondary bile acid[48].

In our study, male mice in the florfenicol group had lower concentrations of secondary bile acids and male mice in the azithromycin group had lower concentrations of all bile acids. Gut microbiota and sex hormones may contribute to this difference. Males in the florfenicol and azithromycin group exhibited lower concentrations of *Bacteroides* and *Lactobacillus*, respectively. These taxa have been associated with bile acid homeostasis, and decreased relative abundance may result in dysfunction of bile acid synthesis[49]. In addition, the sex hormone in males is correlated with bile acid concentration, which might result in a decrease in the excretion of bile acid[50]. The reason for the sex-associated differences in the alteration of bile acids caused by antibiotics is not clear and should be investigated in future studies.

In this study, both azithromycin and florfenicol were observed to increase adipogenesis in mice, and growth was accelerated in male mice, which is supported by most previous studies. For example, both of the antibiotics decreased the relative abundance of *Lactobacillus*, and this was consistent with the finding by M. Cox et al. In their study, when the same amount of food was ingested, the penicillin group with a lower relative abundance of *Lactobacillus* exhibited more weight gain than the control group[51]. We found that the increased relative abundance of *Rumen* caused by azithromycin was associated with adipogenesis in mice. A previous study demonstrated that individuals with a higher abundance of *Rumen* bacteria had a higher incidence of insulin resistance, fatty liver and low-grade inflammation[52]. Azithromycin decreased the relative abundance of *Akkermansia muciniphila*, which could lead to an increase in intestinal inflammation and obesity [53]. The Shannon and Chao index and the body fat measurements of mice in our study also suggested that individuals with low bacterial richness had higher overall body fat than those with high bacterial richness [54,55]. Moreover, some human studies have also found a strong association between antibiotic exposure and obesity in boys[20,23,56,57].

SCFAs and bile acids are important metabolites in fat metabolism. They not only adjust the body's energy intake but also affect fat metabolism [58] [59]. Our study found that body fat rate was significantly higher and the SCFA content was significantly lower in both of the antibiotic groups compared with the control group. The decreased concentration of SCFAs may contribute to obesity as follows. First, because SCFAs can serve as not only a source of energy but also the main nutrients for intestinal cells to maintain intestinal homeostasis, a decrease in SCFAs may lead to nutrition deficiency in intestinal cells and decreased defense, which leads to increased susceptibility to low-level inflammation, eventually leading to the occurrence of obesity [60]. Second, some SCFAs are negatively correlated with the occurrence of obesity. For example, butyric acid and propionic acid can promote gluconeogenesis through the cyclic AMP pathway and brain-gut neural circuits and inhibit the accumulation of fat in adipose tissue[61,62]. Therefore, a decrease in the production of these SCFAs may lead to down-regulation of related metabolic pathways, resulting in the occurrence of obesity.

An increased ratio of primary/secondary bile acids was found in our study, and this increase may lead to obesity via several pathways. First, primary bile acids have been reported to have strong antimicrobial activity and could cause a major shift in gut microbiota composition toward *Firmicutes* and against *Bacteroidetes* [63]. The ratio of *Firmicutes* to *Bacteroidetes* increased significantly in our study, which is a sign of obesity [64,65]. Second, the increase in the primary/secondary bile acid ratio could also lead to a decrease in basal metabolic rate [58,66]. In the case of the same energy intake and daily activities, a lower basal metabolic rate leads to more fat accumulation in the body. In addition to the well-established roles of bile acids in dietary lipid absorption and cholesterol homeostasis, they act as signaling molecules with systemic endocrine functions [67,68]. Alterations in bile acid metabolism can lead to insulin resistance and obesity [43]. For example, bile acids activate mitogen-activated protein kinase pathways and are ligands for the G-protein-coupled receptor TGR5, and secondary bile acids have a much stronger activating capacity for TGR5 than primary bile acids[69]. Down-regulation of TGR5 could disrupt energy metabolism and lead to obesity [70].

## Conclusions

We found that azithromycin and florfenicol altered gut microbiota composition, the production of SCFAs, and the metabolism of bile acids in colonic contents and increased adipogenesis in mice. Some effects showed a sex difference. These findings support the hypothesis that exposure to antibiotics is one important risk factor for childhood obesity, and the effects may be mediated by gut microbiota. More lab studies are needed to investigate specific mechanisms and to confirm the role of gut microbiota in adipogenesis induced by antibiotics.

## Supporting information

S1 Checklist Completed “The ARRIVE Guidelines Checklist” for reporting animal data in this manuscript. (PDF)

S1 Dataset Alpha diversity of the operational taxonomic units for each sample. (CSV)

S2 Dataset Beta diversity for each sample. (TXT)

S3 Dataset Table of OTUs for each sample. (TRE)
